# Markoe on Subcutaneous Perforation of Bone in Ununited Fracture

**Published:** 1859-07

**Authors:** 


### MARKOE ON SUBCUTANEOUS PERFORATION OF BONE IN UNUNITED FRACTURE.

We extract the following notice of drilling from a recent lecture of Dr. Thos. M. Markoe, one of the surgeons of the N. Y. Hospital, for the purpose of showing the result of the employment of the method and the opinions entertained of it in that establishment, and also as showing a change of opinion in regard to the question of priority of its use.

We differ only in some minor points with Dr. Markoe in regard to the value of the method, but as we hope ere long to

---

publish another essay on the subject, we shall not enlarge upon the subject here.

1. *Drilling* the broken extremities, in such a manner as to wound, as far as may be, the opposed surfaces, and thereby to reproduce in a certain degree, the condition of recent fracture. As we now perform it, it was first suggested by Dr. Brainard of Chicago. The operation consists in making, first, a minute puncture through the skin, near the seat of fracture, and then introducing such a drill as this I show you, which is nothing more, in fact, than a long carpenter's drill, and with it boring in various directions, wounding as far as possible the surfaces of the fragments, as often as may seem necessary, to excite some action in their bony tissue. This operation may be repeated every eight or ten days according to the effects produced, or until union is found to be commencing, the parts being kept in the meantime in good apposition and at rest. The principle of this operation is, by the wounding of the broken extremities of the bone, to excite, by that means, anew, the disposition in the parts to throw out reparative material. It is supposed that when, in any case of fracture, the reparative nisus has failed of its effect, the disposition to repair ceases, in a great degree, and even if the obstructing cause be removed, the parts will remain quiescent, until the reparative effort is, in some way, again aroused. This is in fact the principle upon which are founded all the surgical procedures, which have gained any repute, in the management of these cases. This operation by drilling is a simple, easy application of this principle, and its subcutaneous character, while it does not impair its efficiency, renders it less liable to produce evil consequences. In its present form, it is too recent to be precisely appreciated as to its result, but it has been sufficiently tried to show, that in a certain number of cases, it will suffice for a cure. We have employed it in a number of cases, in this hospital, and in some of them, with a very satisfactory success. It has the great merit of being comparatively safe, and in a certain class of cases, not the worst, is a very valuable surgical resource.

